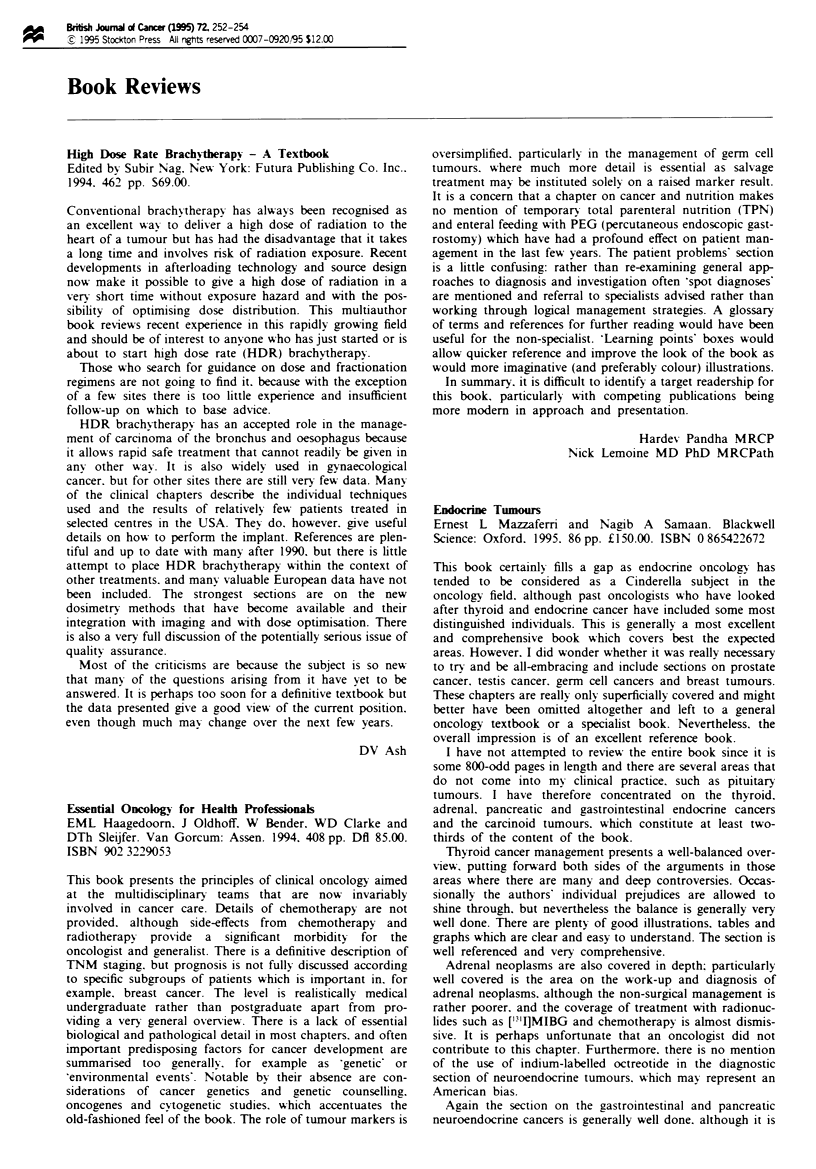# Essential Oncology for Health Professionals

**Published:** 1995-07

**Authors:** Hardev Pandha, Nick Lemoine


**Essential Oncology for Health Professionals**
EML Haagedoorn, J Oldhoff, W Bender, WD Clarke and DTh Sleijfer. Van Gorcum: Assen. 1994, 408 pp. Dfl 85.00. ISBN 902 3229053

This book presents the principles of clinical oncology aimed at the multidisciplinary teams that are now invariably involved in cancer care. Details of chemotherapy are not provided, although side-effects from chemotherapy and radiotherapy provide a significant morbidity for the oncologist and generalist. There is a definitive description of TNM staging, but prognosis is not fully discussed according to specific subgroups of patients which is important in, for example, breast cancer. The level is realistically medical undergraduate rather than postgraduate apart from providing a very general overview. There is a lack of essential biological and pathological detail in most chapters, and often important predisposing factors for cancer development are summarised too generally, for example as 'genetic' or 'environmental events'. Notable by their absence are considerations of cancer genetics and genetic counselling, oncogenes and cytogenetic studies, which accentuates the old-fashioned feel of the book. The role of tumour markers is oversimplified, particularly in the management of germ cell tumours, where much more detail is essential as salvage treatment may be instituted solely on a raised marker result. It is a concern that a chapter on cancer and nutrition makes no mention of temporary total parenteral nutrition (TPN) and enteral feeding with PEG (percutaneous endoscopic gastrostomy) which have had a profound effect on patient management in the last few years. The patient problems' section is a little confusing: rather than re-examining general approaches to diagnosis and investigation often 'spot diagnoses' are mentioned and referral to specialists advised rather than working through logical management strategies. A glossary of terms and references for further reading would have been useful for the non-specialist. 'Learning points' boxes would allow quicker reference and improve the look of the book as would more imaginative (and preferably colour) illustrations.

In summary, it is difficult to identify a target readership for this book, particularly with competing publications being more modern in approach and presentation.

Hardev Pandha MRCP
Nick Lemoine MD PhD MRCPath